# Supplementary material for: Modulation of Macrophage Redox and Apoptotic Processes to Leishmania infantum during Coinfection with the Tick-Borne Bacteria Borrelia burgdorferi
Source: Pathogens. 2023 Sep 4;12(9):1128. doi: 10.3390/pathogens12091128 (PMC10537792; doi:10.3390/pathogens12091128)
Supplement: Supplementary file 1 [file pathogens-12-01128-s001.zip › pathogens-2557159-supplementary.pdf]

THE UNIVERSITY OF IOWA

Re: ANIMAL PROTOCOL [redacted]

The following application submitted to/for US Department of Health & Human Services, NIH

Was reviewed and approved by this institution's Animal Care and Use Committee on **05/02/2022 (Exp. 05/02/2025)**

Title of Application:	[redacted]
Species:	**Dog**
Principal Investigator:	**Petersen, Christine**
Institution:	**The University of Iowa - PHS Assurance No. D16-00009 (A3021-01)**
Identification Number:	

Animals in this study will be utilized in accordance with all PHS policies and the Guide for the Care and Use of Laboratory Animals, NIH Publication No. 85-23, revised 2010.

This institution's Animal Care and Use Committee required no modifications to the above referenced application.

Gwen Waddingham

Gwen Waddingham, IACUC Administrator
University of Iowa Animal Care and Use Commitee

Figure S1: Signed and endorsed animal protocol document of the ethics committee (IACUC)

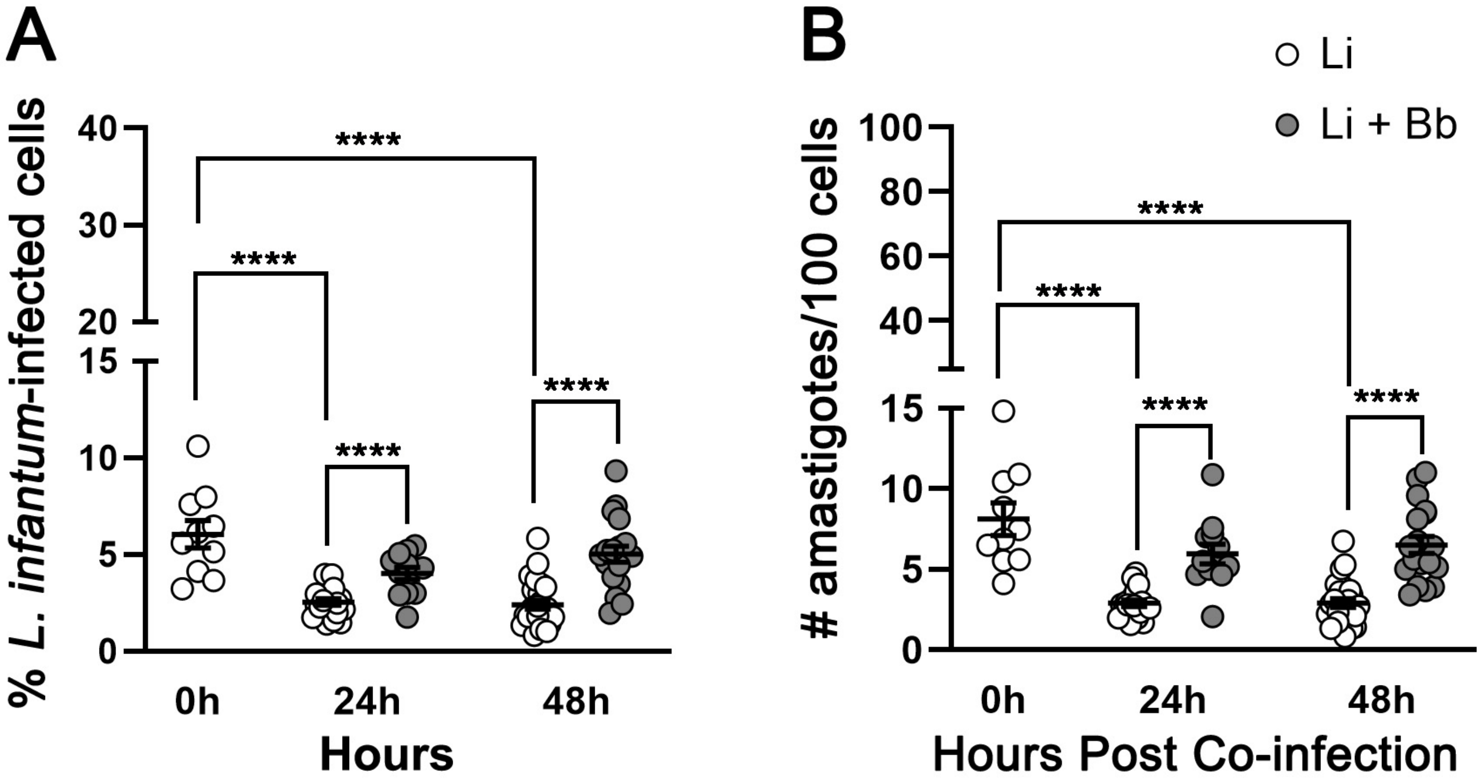

Figure S2. *B. burgdorferi* coinfection increases *L. infantum* infection rates and cellular burden in RAW 264.7 cells. Cells were exposed to *L. infantum* (1:10 MOI) for 24h, and then exposed to live *B. burgdorferi* (1:25 MOI) for indicated time points. Cells were fixed with methanol and stained using HEMA 3 solutions. Frequencies of *L. infantum*-infected cells (A) and number of amastigotes (B) per 100 RAW 264.7 post-coinfections with live *B. burgdorferi* were assessed through light microscopy (100x). Blind and independent assessments were performed by two researchers. Data represent mean ± SEM of three independent experiments, done in triplicates for each condition. *N* = 10-23 coverslips per group. Two-way ANOVA, Tukey's multiple comparisons test. *p***** <0.0001. Li: *L. infantum* single infection. Li + Bb: *L. infantum* and *B. burgdorferi* coinfection.

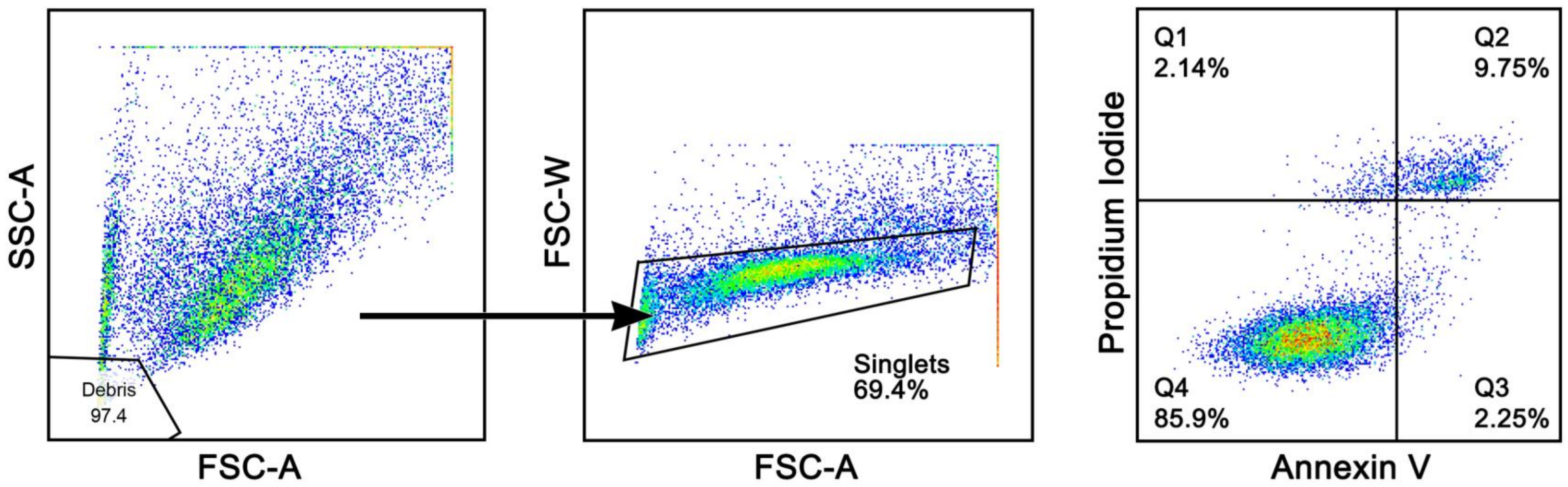

Figure S3. Gating strategy scheme for analysis of apoptosis/necrosis by flow cytometry. DH82 cells were first gated to exclude debris and then followed by a singlet gate based on forward scatter area and width. Live (Q4), apoptotic (Q2 and Q3), and dead singlets (Q1) were discriminated by plotting Annexin V *versus* Propidium Iodide (PI).

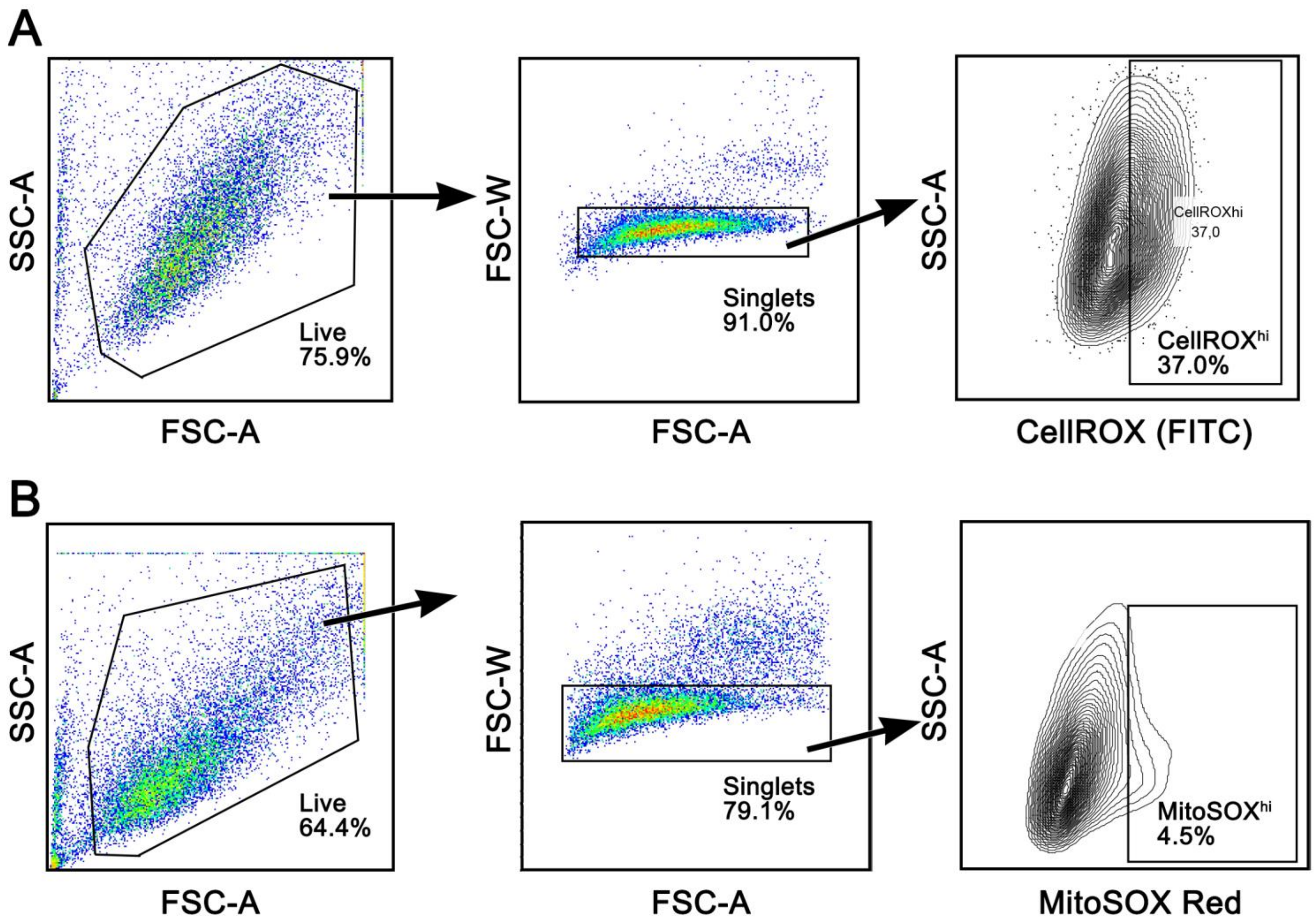

Figure S4. Gating strategy scheme for detecting ROS production by flow cytometry. DH82 cells were first gated to exclude debris as well as dead cells and then followed by a singlet gate based on forward scatter area and width. CellROX^{HI} (A) and MitoSOX^{HI} (B) singlets were discriminated by plotting either FITC or MitoSOX Red *versus* side scatter area.

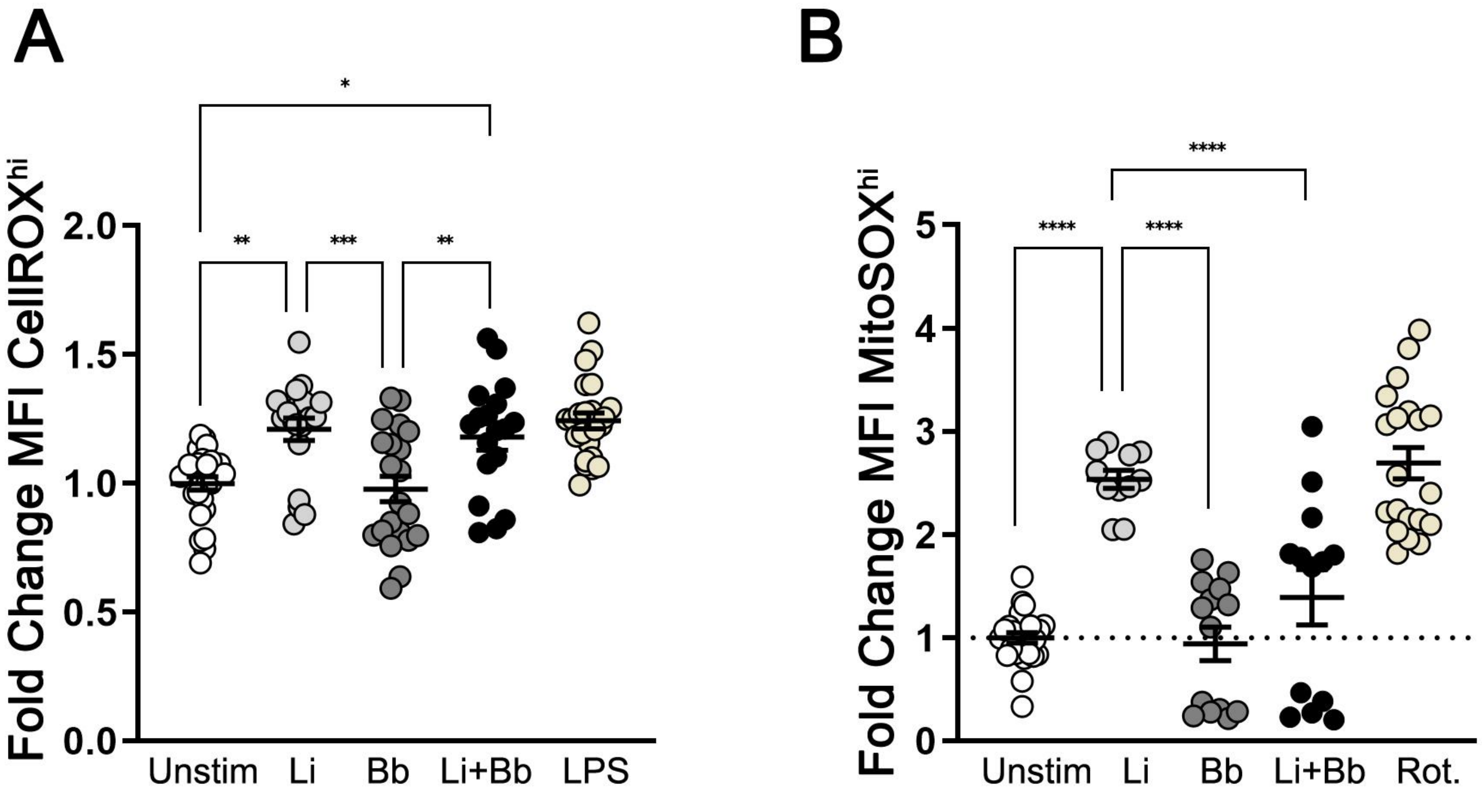

Figure S5. Fold changes in mean fluorescence intensity (MFI) of CellROX^{HI} and MitoSOX^{HI} staining in DH82 cells. Fold change MFI of CellROX^{HI} (A) and MitoSOX^{HI} (B) cells after *B. burgdorferi* exposure and indicated stimulations. The fold increase values were calculated with respect to the average MFI from unstimulated controls. Statistical significance was estimated using ordinary one-way ANOVA with a post-hoc Tukey test. (A) $p^* = 0.0128$; $p^* = 0.0018$ (unstimulated vs. Li), 0.0068 (Bb vs. Li+Bb); $p^{***} = 0.001$; (B) $p^{****} < 0.0001$). Data are presented as mean ± SEM of triplicate experiments. Rot.: rotenone. Li: *L. infantum*-single infection; Bb: *B. burgdorferi*-single infection; Li + Bb: *L. infantum* and *B. burgdorferi* coinfection.

Table S1. Primers sequences used for real-time quantitative PCR.

Gene	Primer sequence (5′ to 3′)	
	**Forward**	**Reverse**
ACTB	ATCCTGCGGCATCCATGAAA	CAGGGGGTGCGATGATCTTG
B2M	GTTTAGCTGCCGTGTAAAGCA	TCAGTTGTCTCGGTCCCACTTA
IFNG	GCGCAAGGCGATAAATGAAC	CTGACTCCTTTTCCGCTTCCT
IL10	CTCCCTGGGAGAGAAGCTCAA	ACAGGGAAAAATCGGTGACA
IL12A	AGGCCTCTTTTATGACGGTCC	AAGCTTTGCGTTCATGGCCT
IL12B	AGGAGAGCCTACCCATCGAG	GGTGGGTCTGGTTTGATGAT
IL17A	CACTCCTTCCGGCTAGAGAA	CACATGGCGAACAATAGGG
IL1B	TACCTGTGGTCTTGGGCATC	TCTAGCTGTAGGGTGGGCTT
IL22	TGCTGGCTAAGGAGGCTAGTT	ACTCCGTGGAACAGTTTCTCC
IL23	CTCACAGAAGCTCTGCACGC	CTCTTCTCTTGG-TAGGTCCACAT
IL6	TTAAGTACATCCTCGGCAAAATCT	CAGTGCCTCTTTGCTGTCTTCA
NOX2	CAAGATGCGTGGAAACTACCTAAGAT	TCCCTGCTCCCACTAACATCA
SOD2	CGCTGGAGAAGGGTGACATT	CACGTTTGATGGCTTCCAGC
TGFB	TACATTGACTTCCGCAAGGA	GTTAGCGTGGTAACCCTTGG
TNFA	TCATCTTCTCGAACCCCAAG	ACCCATCTGACGGCACTATC
